# The case for developing publicly-accessible datasets for health services research in the Middle East and North Africa (MENA) region

**DOI:** 10.1186/1472-6963-9-197

**Published:** 2009-10-29

**Authors:** Shadi S Saleh, Mohamad S Alameddine, Fadi El-Jardali

**Affiliations:** 1 Department of Health Management and Policy, American University of Beirut, Van Dyck Room 111C, PO BOX 11-0236, Riad El Solh, Beirut, 1107 2020, Lebanon

## Abstract

**Background:**

The existence of publicly-accessible datasets comprised a significant opportunity for health services research to evolve into a science that supports health policy making and evaluation, proper inter- and intra-organizational decisions and optimal clinical interventions. This paper investigated the role of publicly-accessible datasets in the enhancement of health care systems in the developed world and highlighted the importance of their wide existence and use in the Middle East and North Africa (MENA) region.

**Discussion:**

A search was conducted to explore the availability of publicly-accessible datasets in the MENA region. Although datasets were found in most countries in the region, those were limited in terms of their relevance, quality and public-accessibility. With rare exceptions, publicly-accessible datasets - as present in the developed world - were absent. Based on this, we proposed a gradual approach and a set of recommendations to promote the development and use of publicly-accessible datasets in the region. These recommendations target potential actions by governments, researchers, policy makers and international organizations.

**Summary:**

We argue that the limited number of publicly-accessible datasets in the MENA region represents a lost opportunity for the evidence-based advancement of health systems in the region. The availability and use of publicly-accessible datasets would encourage policy makers in this region to base their decisions on solid representative data and not on estimates or small-scale studies; researchers would be able to exercise their expertise in a meaningful manner to both, policy makers and the public. The population of the MENA countries would exercise the right to benefit from locally- or regionally-based studies, versus imported and in 'best cases' customized ones. Furthermore, on a macro scale, the availability of regionally comparable publicly-accessible datasets would allow for the exploration of regional variations and benchmarking studies.

## Background

Improving the performance and functioning of health care systems in any country is greatly enhanced by the existence of publically-accessible datasets. These datasets, mostly collected and hosted by governmental agencies or private organizations, are accessible (as data files) either directly from the data keepers or through a review process organized by a host agency (e.g university, research center, etc.). They provide an opportunity for researchers, policy makers and practitioners to easily access relevant information to answer key questions such as assessing the optimal health care policies and its effects; the health status of the population; the amount of money that is being spent on a particular intervention or sector; the number of health workers needed for the delivery of health services and the quality of care delivered among others.

In many developed countries, the availability of publicly-accessible databases has modified the field of public health research in general, and health services research in particular, in innumerable ways. Historically, many entities (e.g. governmental agencies, health care providers, insurance companies, etc.) systematically collected/generated data to serve various functions (e.g. billing and collection, intra-organizational quality measurement and improvement, human resources management, strategic planning, etc.). However, access to these databases was mostly limited to the entity itself and was not made available to external parties. The beginning of the granting of public accessibility to databases trend can be traced to certain types of datasets such as clinical registries, delivery/utilization and financial information, as well as population surveys. In each of these types, there were different purposes for the existence of the various publicly-accessible datasets. Currently, the number and type of publicly-accessible datasets related to health services research are too abundant to take stock of. Furthermore, the recent trend of merging data from various countries (e.g. Organization of Economic Cooperation and Development (OECD), registries, etc.) comprises a unique opportunity for health services researchers to consider performing comparative investigations.

In the Middle East and Northern Africa (MENA) region, as is the case in other developing regions of the world, efforts of governmental and non-governmental agencies to answer many health care services-related questions are often hindered by an assortment of challenges, many of which relate to data sources - scarcity, restrictions on access, incomparability, outdated, etc. These challenges often lead to governments and health analysts relying on inaccurate estimations and forecasts in assessing the performance and planning the future of their health care systems. Inaccurate numbers also shift decision making in the health care field from the realm of health policy to that of politics.

To initiate the process of creating and providing access to datasets, certain preconditions have to exist. These include, but are not limited to, political will, administrative capabilities, stakeholders' buy-ins and demonstrated utility (or good argument for it). Ultimately, the advocacy for the creation and effective utilization of publicly-accessible data repositories should be based on the perceived utility of such datasets in supporting health systems planning and policy making, as well as evaluating the current health care structure and its efficiency. The bullet points below outline some of the potential benefits that could be reaped from creating publically-accessible datasets.

## The potential benefits from the creation of publicly-accessible databases

• ***Ensuring the comparability and quality requirements ***of various data sources by creating and coordinating health information standards [1]

• ***Allowing the analysis of past and forecasting future trends ***through the creating of longitudinal databases on health spending, health delivery and health human resource [2].

• ***Supporting evidence based health planning and decision making ***by providing accurate information and analyses on the performance of health care systems [3].

• ***Building capacity in evidence based research and policy analysis ***by granting researchers access to data sources (with strong protection of privacy and confidentiality of individual information) that could help them answer their research questions [4].

• ***Advancing medical practice by enhancing the understanding on the patterns of health and disease in the population ***through the creation of large longitudinal datasets of patient records [5].

• ***Allowing linkage of datasets ***from different sectors to analyze broader determinants of health [5].

• ***Identifying and promoting national health indicators***. (mortality, morbidity, per capita health spending, etc.) and ensuring regional/international comparability [6].

• ***Enhancing transparency and accountability in health care systems ***through a number of knowledge translation activities (e.g. publications, analyses, educational sessions and conferences) to a variety of stakeholders including patients [7].

• *Allowing the establishment of benchmarks and identification of best practices *in the financing, organization and delivery of health programs [8]; [9].

The advocacy for such utility can actually be supported through evidence. As a start, significant advancement in evidence-based clinical practice resulted from research studies utilizing publicly-accessible datasets, especially clinical registries. Of the many examples in which the use of publicly-accessible datasets advanced evidence-based clinical practice, two are noteworthy: the use of "Statins" ("Statin" is a class of hypolipidemic drugs used to lower cholesterol levels in people with or at risk of cardiovascular disease) in the prevention of cardiovascular and other diseases [10-14] and the potential protective effects of Nonsteroidal Anti-Inflammatory Drugs (NSAIDs) against cancer and other chronic diseases [15-19].

In addition, analysis of large publicly-accessible datasets could also help shape, inform and evaluate the organization, delivery and financing of health services in a health care system. An infamous example is the RAND Health Insurance Experiment (HIE) which presented a persuasive evidence that cost sharing (compared to free health services) reduced the use of health services without necessarily reducing the quality of care provided or causing adverse effect on consumers' health. The experiment itself is an example of the importance of the availability of public datasets. Another example on the central role datasets are playing in shaping health care is NHS's publicly accessible Secondary Uses Services (SUS) dataset which is poised in 2009 to become NHS's single, authoritative and comprehensive repository of high quality data on all aspects of patient care. In recent years, many professional organizations have focused considerable effort on advocating for the use of decision making based on research generated from representative and/or valid datasets. These organizations, e.g. International Society for Pharmacoeconomics and Outcomes Research and AcademyHealth, convened task forces that advocate for such an approach to decision making [20].

The limited existence and poor quality of publicly-accessible datasets in developing countries, especially the MENA region, is a great hindrance to the advancement of evidence-based decision making. In fact, there is a dearth of information when it comes to the degree of availability of such datasets in the Region. In this paper, we aim to provide a scan of what publicly-accessible datasets are available in MENA; we focus on building the case for MENA countries to adopt a strategy of creating and providing access to publicly-accessible datasets as a valuable tool in clinical and policy decision making. Recommendations are also presented that target potential actions by governments, researchers, policy makers and international organizations.

## Publicly-accessible datasets in the MENA context

### Methodological Approach

A four pronged approach was devised in an effort to take stock of what publicly-accessible datasets are available in the MENA region. The search was initiated with an online review of the websites of the regional office of international organizations like the World Health Organization, East Mediterranean Regional Office (WHO-EMRO) in addition to individual country office websites affiliated with WHO. The information collected from this review was supplemented by a review of the websites of the Ministries of Health and Social Affairs of the 24 countries of the MENA region. Compiled information was synthesized by one of the authors and reviewed through a number of research meetings by the research team. If the collected information about any of the MENA countries was deemed inaccurate and/or insufficient to confirm the existence and/or public accessibility of datasets, the Ministries in respective countries were contacted by email and/or phone.

**Figure 1:**
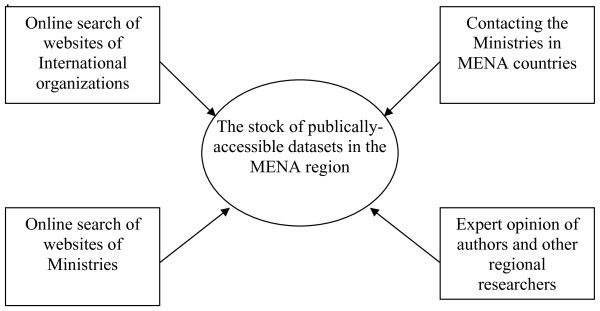
**The methodological approach used to take stock of publically accessible datasets in the MENA region**.

Verification of collected information was done through the triangulation of data from various sources. It was further strengthened by the informed opinion of the authors and experts with considerable work and research experience in the Region (Figure 1).

### Overview of Publicly-accessible Datasets in MENA

Our search revealed that datasets in the MENA region generally do not exist in an open-access format. Some datasets are housed in professional organizations (e.g. orders or syndicates of physicians, nurses) or Ministries, yet is only available in aggregate format (e.g. reports drafted by organizations or Ministries) - Table 1. This data can be compiled into spreadsheets; however, lack of accessibility remains a major obstacle throughout the region.

**Table 1:** An overview of dataset availability in the MENA region

	**MENA Countries**	**Human Resources in Health**	**Health Data**	**Quality**	**Health System and facilities**	**Financial Statistics**	**Middle East Cancer Consortium^¥^**	**Middle East IVF Registry^£^**
1	Afghanistan	✓	✓	✓				

2	Bahrain	✓	✓		✓	✓		✓

3	Cyprus		✓				✓	

4	Djibouti ^(F)^	✓						

5	Egypt	✓	✓				✓	✓

6	Iran	✓	✓	✓				

7	Iraq	✓	✓					

8	Jordan	✓	✓				✓	✓

9	Kuwait	✓	✓					

10	Lebanon	✓	✓			✓		✓

11	Libya	✓	✓					

12	Morocco ^(F)^	✓	✓					

13	Oman	✓	✓					

14	Palestine*	✓	✓		✓		✓	

15	Pakistan	✓	✓					

16	Qatar	✓	✓		✓			

17	Saudi Arabia	✓	✓					✓

18	Somalia	✓	✓		✓			

19	Sudan	✓	✓		✓			

20	Syria	✓	✓					

21	Tunisia ^(F)^	✓	✓					✓

22	United Arab Emirates	✓	✓					✓

23	Western Sahara**		✓					

24	Yemen	✓	✓		✓			

* Referred to as West Bank and Gaza on some international websites; This is the only MENA country with publicly accessible databases (through the (Palestinian Central Bureau of Statistics)

** This is a conflict country

¥ Lebanon is not a member of this consortium, probably since Israel is also a member: . They have an 82-page "Manual of standards for Cancer Registration" available on:  They also have a link to all publications from the registry on: ;

£ No website was found for the In-Vitro Fertilization registry. A report by Ragaa Mansour about the IVF registry was documented

Human Resources for Health (HRH) datasets were available for all countries in MENA region (with exception of Cyprus and Western Sahara). Such datasets included absolute numbers and densities of physicians, nurses, midwives, dentists, pharmacists, physiotherapists, medical assistants, clinical officers, laboratory personnel, radiologists, environmental and public health workers, community health workers, administrative and support staff, and miscellaneous technicians. It should be noted that many of the above health professional categories are further divided into technicians or assistants. However, most countries did not report all of the above data on an annual basis, with the exception of Pakistan which had the most detailed results. The data source for most of these countries was the HRH observatory for the WHO-EMRO [21]. Other data sources included the ministries of health websites.

Findings of the search revealed that quality of care data is sparse for countries in the region. The only data available were from Afghanistan and Iran. Documentation of a balanced score card initiative was found for Afghanistan for 2006 and 2007 [22]. As for Iran, the WHO Country Office has 32 programs which include health management information system (HMIS), health policy development, health system development, and health system research, among other topics [23] (Table 1).

Health system facilities data was available for some countries in the region. For example, Bahrain has a detailed ministry of health (MOH) website which included data on financial statistics (total government expenditure, MOH expenditure, share of health expenditure by government, and revenues to ministry by fiscal year [1998 to 2007]) [24]. As for the case of Qatar, most of the information available on the MOH website included data on health systems and facilities like hospitals, pharmacies and health care centers (by geographic location) [25]. The only information available on health systems in Somalia included laboratory support - mostly as it relates to Hepatitis B and Tuberculosis [26]. Sudan is trying to improve the health status of its population through the establishment and rehabilitation of health facilities, yet collected heath information primarily focused on Darfur [27]. As for the case of Yemen, some information was found on distribution of health facilities [28].

Similar to the case of quality, information on health finances statistics was limited to only two countries; Bahrain and Lebanon. In Bahrain, the reports of annual statistics dating from 2000 to 2007 included some financial data (total government expenditure, MOH expenditure, share of health expenditure by government, and revenues to ministry by fiscal year [1998 to 2007]) [24]. In Lebanon, the only information relating to this issue on the MOH website was the number of granted "visas" for inpatient visits for 2004. According to the Lebanese MOH, a visa refers to granted approval for hospitalization at the MOH expense for eligible patients. The Lebanese MOH has recently compiled a database on clinical utilization and spending indicators, which include data for many but not all hospitals. These variables include: total inpatient cost per hospital, total admissions and days of admission, total one-day admissions, paid cost per day, repeat diagnosis, emergency admissions, ICY admissions, and rates for specific conditions such as Cataracts, Cardiology, Cancer and Chemotherapy. The data can also be used to derive a relative clinical risk and relative efficiency scores. However, not all hospitals regularly collect this data which could be used by the MOH for benchmarking and performance contracting [29].

In short, despite of the availability of certain types of data related to health systems in the region, our findings clearly show that such data is mostly presented in a report format in addition to not being consistently available across MENA countries. However, one exception is noteworthy as our search revealed that the Palestinian Central Bureau of Statistics provides public access to a number of datasets based on relatively recent data (e.g. 2007 census). The datasets available cover a number of health topics and studies such as access to health services, demographic and health survey, finance and insurance survey, health providers and beneficiaries survey, and household health expenditure survey among many others. All studies and topics have brochures, a code book and the questionnaire. It should be noted that the datasets are available for a small fee for individuals, local institutions and international institutions [30].

**Figure 2:**
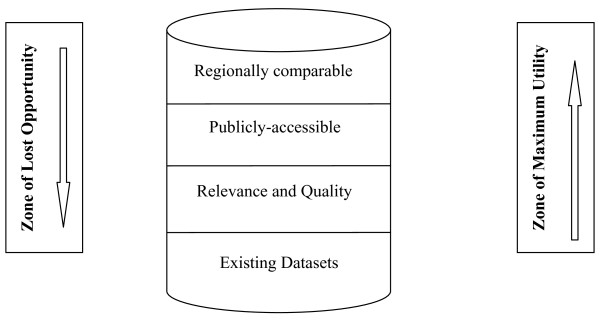
**A Conceptual Illustration of Maximizing the Utility of Datasets**.

However, except the success story of the Palestinian Central Bureau of Statistics, our overview of dataset availability in the MENA region revealed that the solution to the problem of modest availability of publicly-accessible datasets should be gradual and start from ensuring availability to relevance and usefulness (quality), accessibility and comparability. This stems from the fact that there may be a wealth of datasets available in the region, yet the relevance and usability of these datasets is dubious in most instances. When usable datasets are available, they are seldom accessible to researchers and policy makers. Finally, little is done to ensure regional comparability of available datasets across the MENA countries. Figure 2 presents a graphical illustration of how to maximize the utility of datasets.

## Discussion

The existence of publicly-accessible datasets comprised a significant opportunity for health services research to evolve into a science that supports health policy making and evaluation, proper inter- and intra-organizational decisions and optimal clinical interventions. However, most of these developments in the discipline occurred in the US and Europe. Other parts in the World, specifically the MENA region, did not witness a parallel evolvement in publicly-accessible datasets. Most of the datasets in the region remain private or have strict guidelines for accessibility and use. As such, this paper is a call for all policy makers and health services researchers in the region to actively start lobbying for the public availability and accessibility of the datasets that would help explore/solve the many health care issues that plague the region.

For example, clinical registries are an important resource that countries in the MENA region could tap into to monitor, assess and advance the health of their population. The uses of registries are many and their potential is outstanding, especially if MENA region countries were able to link information collected across different countries. This would allow the comparison of diseases incidence rates across regions, populations and countries. In addition, its linkage with other datasets (e.g. claims) can produce valuable evidence for policy planning and identifying cost-effective treatments. Similarly, longitudinal analysis of human resource registration databases could help enhance the understanding of researchers and policy makers on employment patterns and attrition rate from the workforce. This would help inform future resource planning and optimize matching the demand and supply of health human resources. This is pivotal considering the global shortage of health human resources, which is intensified in the MENA region.

Based on this, the MENA region has a prime opportunity to join the trend of evidence-based decision making. Specifically, we think the following recommendations comprise actionable steps:

• Lobbying Ministries of Health (and Social Affairs where applicable) to take stock of what datasets are available. In addition, plan and implement strategies to highlight their existence and methods to access these datasets

• Encouraging of governmental health agencies, specifically Ministries of Health, to play a central role in employing - and advocating for - evidence-based policy making

• Advocating for international and regional entities to act as coordinators for data banks, as well as support for data building infrastructure projects

• Highlighting the role academic institutions and national statistics bodies can play as independent parties for data collection, storage and analysis

• Advising and strengthening of regional efforts to standardize collected data as much as possible

• Establishing a higher committee within each country - as well as regionally - that would identify a minimum dataset for targeted areas of data collection

• Encouraging regional researchers to focus on relevant research topics and highlight the importance of the findings to policy makers and practitioners

• Highlighting regional knowledge exchange around best practices and comparative studies

It is our belief that such steps would form a concrete foundation for the establishment of a friendly environment for the creation of publicly-accessible datasets. The start of the process can/should be modest. For example, taking stock of the available datasets and exerting an effort to make what is available, or at least part of it, public (or simplifying the process of gaining access to the raw data).

This paper has limitations that merit consideration. First, the WHO website has a link to individual country profiles for all countries, but information is not always complete, up-to-date or accurate. This data cannot be considered a registry, but the WHO-SIS documents data on a semi-annual basis. Second, while the search conducted included all countries of the MENA and most accessible data sources, it may not have been comprehensive. In this regard, it should be acknowledged that not all data sources in the region are publicly accessible to researchers or the general public. Some databases, registries and even reports may require certain organizational privileges.

In conclusion, policy makers in this region should be ready and willing to base their decisions on solid representative data and not on estimates or small-scale studies; Researchers should be able to exercise their expertise in a meaningful manner to both, policy makers and the public. In addition, researchers have the responsibility to highlight the utility of evidence-based decision making as a reason for increasing public accessibility of data. Finally, the population of the MENA countries has the right to benefit from locally- or regionally-based studies, versus imported and in best cases customized ones that have a direct effect on the clinical care they receive and as the basis for policies which affect their health care. We acknowledge that what we are calling for here might be a paradigm shift for a number of countries in the MENA region. We do not anticipate change to happen overnight but suggest a course of action that if taken could bring a lot of benefit to the region.

## Summary

• Publicly-accessible datasets represent a catalyst for the enhancement of health care systems through the ability to conduct policy- and clinically-relevant health services research

• Our search revealed that although datasets are available in most Middle East and North African (MENA) countries, they are limited in terms of quality, accessibility and relevance which hinders the ability to explore the datasets' full potential

• Such finding represents a lost opportunity for the evidence-based advancement of health systems in the region

• Practical recommendations targeted towards researchers, policy makers and agencies (public and private) are presented to make the case for the development of relevant, accessible and regionally-comparable datasets

• The move towards the wide-spread of publicly-accessible datasets will have to be an incremental process and will depend upon each of the countries' existing infrastructure and willingness to incorporate such an agenda in strategic planning for health

## Competing interests

The authors declare that they have no competing interests.

## Authors' contributions

SS conceptualized the idea for the paper, oversaw its development, critically contributed to the drafting and revision of the paper and has given final approval of the version to be published. MA contributed to the concept and design of the paper, critically contributed to the role of publicly-accessible datasets in developing countries section and revision of the paper and has given final approval of the version to be published. FJ contributed to the concept and design of the paper, examined the landscape of the existing publicly-accessible datasets in the region, revised of the paper and has given final approval of the version to be published.

## Pre-publication history

The pre-publication history for this paper can be accessed here:


